# Operative and nonoperative management of acute cholecystitis in patients on chronic kidney replacement therapy

**DOI:** 10.1002/jhbp.12133

**Published:** 2025-03-25

**Authors:** Dharmenaan Palamuthusingam, Carmel M. Hawley, Elaine M. Pascoe, David Wayne Johnson, Palvannan Sivalingam, Simon T. Wood, Pranavan Palamuthusingam, Matthew D. Jose, Magid Fahim

**Affiliations:** ^1^ Metro North Kidney Health Service Royal Brisbane and Women's Hospital Brisbane Queensland Australia; ^2^ Faculty of Medicine University of Queensland Brisbane Queensland Australia; ^3^ School of Medicine Griffith University Southport Queensland Australia; ^4^ Metro South Integrated Nephrology and Transplant Services Princess Alexandra Hospital Brisbane Queensland Australia; ^5^ Australasian Kidney Trials Network (AKTN) University of Queensland Brisbane Australia; ^6^ Centre for Health Services Research University of Queensland Brisbane Queensland Australia; ^7^ Metro South Kidney and Transplant Services Princess Alexandra Hospital Brisbane Queensland Australia; ^8^ Translational Research Institute Brisbane Queensland Australia; ^9^ Department of Anaesthesia Princess Alexandra Hospital, Metro South Health Service Brisbane Queensland Australia; ^10^ Department of Urology Princess Alexandra Hospital, Metro South Health Service Brisbane Queensland Australia; ^11^ Department of Surgery Royal Brisbane and Women's Hospital Brisbane Queensland Australia; ^12^ Department of Nephrology Royal Hobart Hospital Hobart Tasmania Australia; ^13^ School of Medicine University of Tasmania Hobart Tasmania Australia; ^14^ Metro South Integrated Kidney and Transplant Services Princess Alexandra Hospital Brisbane Queensland Australia; ^15^ Metro North Health Service Brisbane Queensland Australia

**Keywords:** acute cholecystitis, cholecystectomy, chronic dialysis, kidney failure

## Abstract

**Background:**

Patients with kidney failure receiving chronic kidney replacement therapy (KRT: dialysis or kidney transplantation) have increased risks of postoperative mortality and morbidity. This study assesses the outcomes of acute cholecystitis in patients on chronic KRT who undergo cholecystectomy compared to nonoperative management.

**Methods:**

This bi‐national population cohort study evaluated all incident and prevalent patients receiving chronic KRT using linked data between Australia and New Zealand Dialysis and Transplant (ANZDATA) Registry and jurisdictional hospital admission datasets between 2000 and 2015. Patients with a primary diagnosis of acute cholecystitis were identified using the International Classification of Diseases (ICD) and were divided into two groups: patients who underwent cholecystectomy and those who received nonoperative management. Comorbidity‐adjusted Cox models were used to determine the associations of cholecystectomy with 30‐day and 12‐month mortality.

**Results:**

From the 46 779 patients on chronic KRT, there were 1520 patients with an initial emergency presentation of acute cholecystitis, of whom 87% received nonoperative management. Thirty‐day mortality risk was no different between the two groups (5.4 vs. 5.1%, *p* = .83). Despite higher odds for nonfatal outcomes including composite cardiovascular complications (MI, CVA, cardiac arrest: OR 2.08, 95% CI (1.13–3.81)), ICU admission (OR 3.51, 95% CI (2.41–5.10)), and blood transfusions (OR 2.29, 95% CI (1.60–3.27)), surgery was associated with improved survival at 12 months compared with nonoperative management (HR 0.61, 95% CI (0.43–0.87)). Patients who received nonoperative management had a higher 30‐day readmission rate (17.6 vs. 12.5%, *p* = .44).

**Conclusions:**

In patients with acute cholecystitis, compared with nonoperative management, surgery was associated with better survival at 12 months but higher rates of early morbidity.

## Graphical abstract

Assessing the outcomes of acute cholecystitis in patients on chronic kidney replacement therapy, Palamuthusingam and colleagues found that cholecystectomy did not improve 30‐day survival compared with nonoperative management. Although surgery resulted in higher rates of early morbidity, it was associated with significantly better survival at 12 months.

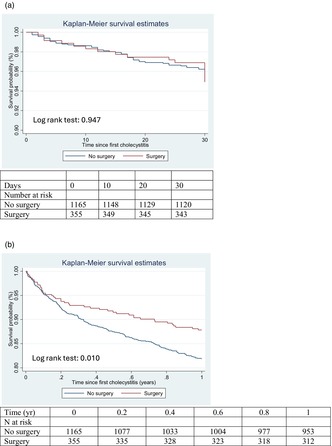

## 1 BACKGROUND

Acute cholecystitis is a common disease in the general population, with early surgical intervention (i.e., cholecystectomy) being associated with lower mortality and morbidity, including reduced intensive care admissions and hospital readmission rates.^1^ However, uncertainty remains about the appropriateness of early surgical intervention compared to initial nonoperative management, including less invasive procedures such as the use of percutaneous cholecystostomy tubes (PTC), in patients considered to have a high perioperative risk such as those patients receiving chronic kidney replacement therapy (KRT) for kidney failure. Chronic KRT refers to hemodialysis (HD), peritoneal dialysis (PD), home hemodialysis (HHD), and kidney transplantation.

A 15‐year binational data‐linkage study in Australia and New Zealand involving 46 779 patients on chronic KRT found that cholecystectomies were not infrequent (0.5 surgeries per 100‐patient years) with an overall 30‐day mortality risk of 3.2%.^2^ The risk of postoperative myocardial infarction was 2.8%, but the risks of a composite of infective complications and 30‐day readmission were 15.8% and 13.3%, respectively.^2^ In the face of these risks, it is possible that certain patients may benefit from initial nonoperative management. However, no previous studies have evaluated the short and long‐term outcomes of operative versus nonoperative management of patients on chronic KRT following their initial presentation with acute cholecystitis.

This study aimed to define the prevalence of acute cholecystitis in patients on chronic KRT, describe current practice patterns in the management of acute cholecystitis, and determine short‐ and long‐term (12‐month) outcomes of patients who do and do not have surgery following their initial presentation.

## 2 METHODS

### 2.1 Study design, data sources, and population

All incident and prevalent patients receiving chronic KRT between July 1, 2000 and December 31, 2015, as identified by the Australia and New Zealand Dialysis and Transplant (ANZDATA) Registry, were linked with jurisdictional hospital admission datasets across Australia and New Zealand using probabilistic linkage for all Australian jurisdictions and deterministic linkage for New Zealand. Probabilistic linkage is a method that uses identifiable personal variables such as first and last name, date of birth, sex, home address, hospital, and medical record numbers to calculate the likelihood that a specific hospital admission is linked to a particular individual. This approach assigns probability weights to each variable, enabling the identification of matching records across different datasets. On the other hand, deterministic linkage was conducted using the National Health Index Number (NHI), a unique identifier assigned to every New Zealand resident by the Ministry of Health.

ANZDATA is a clinical quality registry that records patient demographic, clinical, and KRT modality characteristics on all patients receiving chronic KRT, typically on the 31st of December of each year. Date of death and cause were obtained from ANZDATA. Hospital admission datasets contained clinical information, including medical diagnoses and comorbidities, as defined by the International Classification of Diseases–10 Australian Modification (ICD‐10AM), and surgeries performed during a hospital stay, as defined by the Australian Classification of Health Interventions (ACHI) procedure codes.^3^, ^4^, ^5^ The Strengthening the Reporting of Observational Studies in Epidemiology (STROBE) and Reporting studies Conducted using Observational Routinely collected Data (RECORD) recommendations were followed.^6^, ^7^

### 2.2 Exposure

Hospital admissions for acute cholecystitis were identified when the relevant ICD‐10AM code for acute cholecystitis was present as the primary diagnosis in the index admission (Table S1). Patients' first emergent presentations with acute cholecystitis were analyzed. Patients who underwent cholecystectomies or PTC for their initial emergent presentations with acute cholecystitis were identified using the relevant ACHI codes (Table S2). Patients were divided into two groups: those who presented with acute cholecystitis and underwent cholecystectomy during the index admission (surgery) and those who received nonoperative management for their acute presentation (no surgery). Patients who developed acute cholecystitis during their hospital admission, that is, a secondary diagnosis, and elective admissions were not included.

### 2.3 Covariates

Patient comorbidities were designated at the time of surgery by using a lookback period of patient records to ensure coding reliability, rather than relying on a the single index admission.^8^ Comorbidities extracted included diabetes mellitus (DM), hypertension, congestive cardiac failure (CCF), ischaemic heart disease (IHD), peripheral vascular disease (PVD), cerebrovascular disease (CVD), chronic obstructive pulmonary disease (COPD), and obstructive sleep apnoea (Table S3).

### 2.4 Outcomes

The primary outcome measured was 30‐day all‐cause mortality. Secondary outcomes included postoperative nonfatal complications defined by ICD‐10AM: myocardial infarction (MI), cerebrovascular accident (CVA), cardiac arrest, pulmonary embolism (PE), deep vein thrombosis (DVT), sepsis, mechanical wound complication, surgical site infection (SSI), pneumonia, blood transfusions, delirium, hyperkalaemia, pulmonary edema, and dialysis access intervention. Lengths of stay, intensive care admissions (ICU), and all‐cause 30‐day readmission rates were also measured (Table S4). Hospital readmissions for cholecystitis within 12 months were calculated, and the perioperative outcomes in patients who underwent surgery in these subsequent admissions were also reported.

### 2.5 Statistical analyses

Baseline characteristics of the two cohorts (surgery vs. nonoperative) were described with medians and interquartile range (IQR) for continuous variables, and both counts and percentages for categorical variables. Univariable analysis was performed with the *X* ^2^ test for categorical data, unpaired *t*‐test for normally distributed data, and Wilcoxon rank sum test for non‐normally distributed data. Multivariable Cox proportional hazard models were used to calculate adjusted hazard ratios (HR) for 30‐day mortality. This model was adjusted for age (years), body mass index (BMI; kg/m^2^), KRT vintage (length of time since commencing KRT in years), KRT modality (HD, PD, HHD, or kidney transplant), presence of diabetes mellitus, and ischaemic heart disease. Multivariable logistic regression was undertaken to identify the odds of nonfatal postoperative outcomes during the index admission. Kaplan–Meier survival curves at both 30 days and 12 months were generated.

**FIGURE 1:**
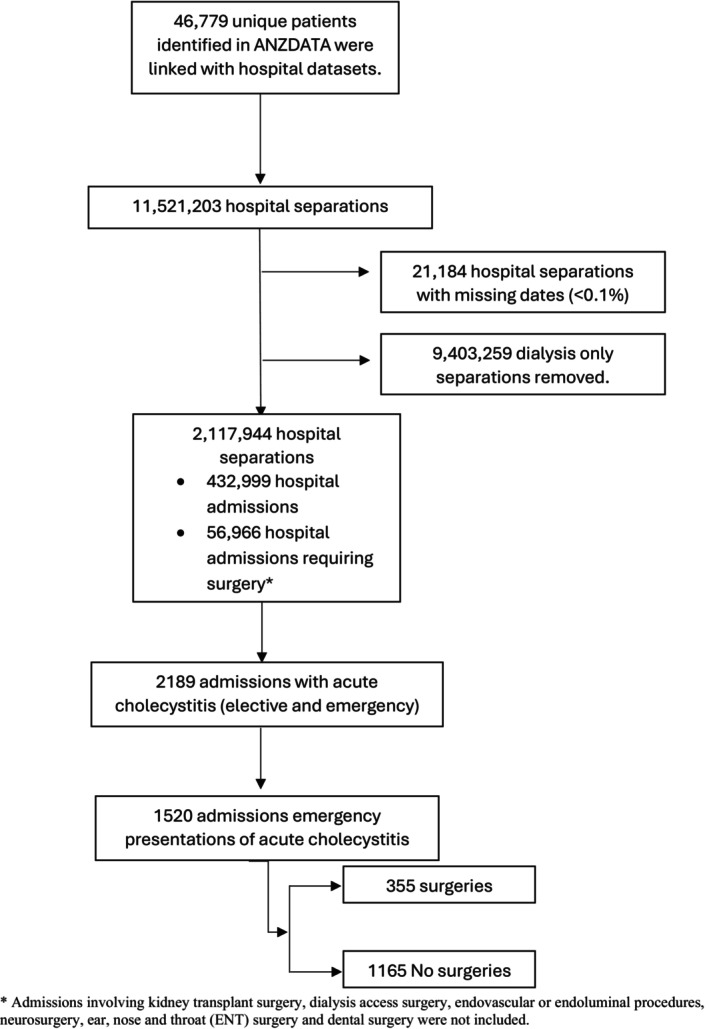
Cohort flow diagram.

In a secondary analysis, both fatal and nonfatal postoperative outcomes for patients on chronic dialysis (HD, PD, and HHD) and kidney transplant recipients were evaluated separately. Statistical analysis was undertaken with Stata version 14.2 (Stata Corporation, College Station, Texas, USA).

### 2.6 Ethics approval

Ethics approval for this study was provided by Human Research Ethics Committees in each of the health jurisdictions involved: New South Wales (HREC/17/CIPHS/41), Queensland (HREC/17/QPAH/636), South Australia (HREC/17/SAH/115), South Australia Aboriginal Health and Research Council (HREC 04‐17‐746), Northern Territory (HREC: 2017‐2962), Department of Health Western Australia (RGS0000000740), Western Australia Aboriginal Health Ethics Committee (HREC: 835), Victoria (HREC/17/QPAH/638—Victoria Specific Module under National Mutual Acceptance memorandum), and Tasmania (H0017537).

## 3 RESULTS

### 3.1 Baseline characteristics

From the 46 779 patients on chronic KRT who were linked between the datasets, there were 2189 individuals who had documented acute cholecystitis as a primary diagnosis, of whom 1520 were categorized as an emergency presentation (Figure 1). Twenty‐three percent (*n* = 355) of patients who presented as emergencies underwent a cholecystectomy during their initial visit. Only 1.6% (*n* = 19) of patients in the nonsurgical group received a PTC (Table 1).

**TABLE 1:** Baseline characteristics.

	Surgery	No surgery			
	*N* = 355	*N* = 1165	*p*‐Value	No percutaneous cholecystostomy, *N* = 1146	Percutaneous cholecystostomy, *N* = 19
Age, years [IQR]	61.7 [50.7–78.5]	62.2 [51.7–72.0]	.326	62.0 [51.3]	71.1 [64.5–85.4]
Gender, male (%)	185 (52.1)	609 (52.2)	.655	595 (57.2)	14 (73.7)
Body mass index (kg/m^2^)	27.3 [20.4–32.9]	27.0 [23.3–31.8]	.409	27.0 [23.3–31.8]	27.8 [24.4–37.2]
Ethnicity					
White	240 (67.6)	647 (55.5)	.007	636 (55.5)	11 (58.0)
First nations Australian	37 (10.1)	180 (15.5)		179 (15.6)	1 (5.3)
Māori	31 (8.7)	148 (12.7)		147 (12.8)	1 (5.3)
Pasifika	18 (5.1)	86 (7.4)		80 (7.0)	6 (31.2)
Asian	25 (7.0)	87 (7.5)		87 (7.6)	–
Other	4 (1.1)	17 (1.5)		17 (1.5)	–
Cause of kidney failure					
Diabetes mellitus	129 (36.3)	427 (36.7)	.375	420 (36.7)	7 (36.8)
Hypertension	27 (7.6)	110 (9.4)		106 (9.3)	4 (21.1)
Glomerulonephritis	110 (31.0)	303 (26.0)		299 (26.1)	4 (21.1)
Reflux nephropathy	24 (6.8)	43 (3.7)		43 (3.8)	–
ADPKD	13 (3.7)	79 (6.8)		77 (6.7)	2 (10.5)
Other	52 (14.7)	203 (17.4)		201 (17.5)	2 (10.5)
Kidney replacement therapy modality					
Hemodialysis	170 (47.9)	682 (58.5)	.441	669 (58.4)	13 (68.4)
Peritoneal Dialysis	48 (13.5)	216 (18.5)		215 (18.8)	1 (5.3)
Home Hemodialysis	24 (6.8)	62 (5.3)		59 (5.2)	3 (15.8)
Kidney transplant	113 (31.8)	205 (17.6)		203 (17.7)	2 (10.5)
Dialysis vintage	4.0 [1.4–9.1]	2.9 [0.6–6.7]	<.001	2.8 [0.5–6.7]	3.1 [1.4–7.3]
Comorbidities					
Ischaemic heart disease	85 (24.0)	343 (29.4)	.044	335 (29.2)	8 (42.1)
Cardiomyopathy/Heart failure	52 (14.6)	242 (20.8)	.181	237 (20.7)	5 (26.3)
Peripheral vascular disease	25 (7.0)	99 (8.5)	.381	99 (8.6)	0 (0)
Cerebrovascular disease	15 (4.2)	65 (5.8)	.318	64 (5.6)	0 (0)
Diabetes mellitus	173 (48.7)	612 (52.5)	.210	600 (52.4)	12 (63.2)
Hypertension	281 (79.2)	934 (80.2)	.676	915 (79.8)	19 (100)
Chronic obstructive airway disease	16 (4.5)	80 (6.9)	.109	79 (6.9)	1 (5.3)
Obstructive sleep apnoea	6 (1.7)	2 (0.2)	.942	35 (3.1)	2 (10.5)

Patient who underwent cholecystectomy was of similar age and BMI to the nonsurgical group (61.7 vs. 62.2 years and 27.3 vs. 27.0 kg/m^2^, respectively), but had been on KRT longer (4.0 vs. 2.9 years, *p* < .001). There were no significant differences in the prevalences of comorbidities between the two groups (Table 1).

### 3.2 Short‐term postoperative outcomes

#### 3.2.1 30‐day mortality: Surgery versus nonsurgical group

**FIGURE 2:**
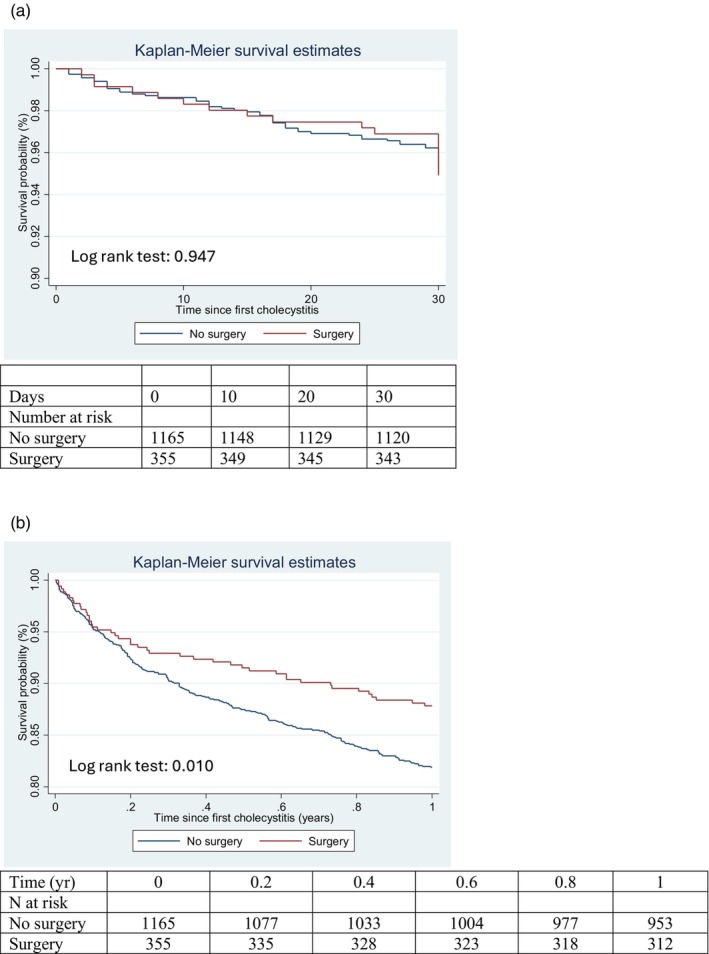
(a) Kaplan–Meier survival curve for 30‐day survival following initial presentation with acute cholecystitis. (b) Kaplan–Meier survival curve for 1‐year survival following initial presentation with acute cholecystitis.

There was no difference in 30‐day mortality between the two groups (5.4 vs. 5.1%, *p* = .830, Table 2). Surgery was not associated with an increased risk of death at 30 days compared to a nonoperative approach (HR 1.33, 95% CI 0.78–2.30, Figure 2a). When stratified by KRT modality, the risk of 30‐day all‐cause mortality was not significantly different between surgical and nonoperative management for patients on chronic dialysis (HR 1.40, 95% CI 0.78–2.54) and for kidney transplant recipients (HR 1.15, 95% CI 0.25–5.35, Table S5).

**TABLE 2:** Posttreatment outcomes (cholecystectomy vs nonoperative).

	Surgery, *N* = 355	No surgery, *N* = 1165	*p*‐Value	No surgery, no percutaneous cholecystostomy, *N* = 1146	No surgery, yes percutaneous cholecystostomy, *N* = 19	*p*‐Value
Death (*n*) % (95% CI)	19 5.4 (3.8–6.3)	59 5.1 (3.8–6.3)	.830	56 4.9 (3.6–6.1)	3 15.8 (0.0–3.2)	.044
Myocardial infarction (*n*) % (95% CI)	9 2.5 (0.9–4.2)	25 2.2 (1.3–3.0)	.664	21 1.8 (1.0–2.6)	4 21.1 (2.7–39.4)	<.001
Stroke (*n*) % (95% CI)	4 1.1 (0.0–2.2)	4 0.3 (0.0–0.7)	.092	4 0.3 (0.0–0.6)	0 (0)	–
Cardiac arrest (*n*) % (95% CI)	8 2.3 (0.7–3.8)	7 0.6 (0.1–1.0)	.010	7 0.6 (0.2–1.0)	0 (0)	–
Pulmonary Embolism (*n*) % (95% CI)	1 0.3 (0.0–0.1)	2 0.2 (0.0–0.1)	.686	2 0.2 (0.0–0.7)	0 (0)	–
Deep Vein Thrombosis (*n*) % (95% CI)	3 0.9 (0.0–0.2)	3 0.3 (0.0–0.1)	.145	3 0.3 (0.0–0.6)	0 (0)	–
Pneumonia (*n*) % (95% CI)	15 4.2 (2.1–6.3)	41 3.5 (2.5–4.6)	.537	39 3.4 (2.4–4.5)	2 10.5 (0.0–24.3)	.115
Surgical site infection (*n*) % (95% CI)	12 3.4 (1.5–5.3)	–	–	–	0 (0)	–
Sepsis (*n*) % (95% CI)	32 11.6 (7.8–15.4)	81 8.3 (6.6–10.1)	.095	74 7.7 (6.0–9.4)	7 43.8 (19.4–68.1)	<.001
Delirium (*n*) % (95% CI)	7 2.0 (0.5–3.4)	22 1.9 (1.1–2.7)	.920	18 1.6 (0.9–2.3)	4 21.1 (2.7–3.9)	<.001
Mechanical wound complication (*n*) % (95% CI)	20 5.6 (3.2–8.0)	–	–	–	1 5.3 (0.0–15.4)	–
Blood transfusion (*n*) % (95% CI)	62 17.5 (13.5–21.4)	106 9.1 (7.4–10.8)	<.001	101 8.8 (7.2–10.5)	5 26.3 (6.5–46.1)	.014
Pulmonary edema (*n*) % (95% CI)	19 4.2 (2.1–6.3)	63 4.2 (3.1–5.4)	.987	61 4.1 (3.0–5.2)	2 10.5 (0.0–24.3)	.184
Hyperkalemia (*n*) % (95% CI)	22 6.8 (4.1–9.6)	45 4.0 (2.9–5.2)	.039	43 3.9 (2.8–5.1)	2 11.1 (0.0–25.6)	.144
Dialysis access dysfunction (*n*) % (95% CI)	16 4.5 (2.3–6.7)	20 1.7 (1.0–2.5)	.004	19 1.6 (0.9–2.4)	1 5.3 (0.0–15.3)	.257
Length of stay [IQR]	9 [5–16]	5 [3–10]	<.001	5 [3–9]	23 [8–48]	<.001
Intensive Care Unit admission (*n*) % (95% CI)	68 19.2 (15.1–23.2)	75 6.4 (5.0–7.8)	<.001	71 6.2 (4.8–7.6)	4 21.1 (2.7–39.4)	.015
Readmission within 30 days (*n*) % (95% CI)	40 12.5 (3.1–21.9)	163 17.6 (9.5–25.8)	.436	161 17.6 (9.5–25.6)	2 10.5 (1.1–21.7)	.114
12‐month deaths (*n*) % (95% CI)	46 12.7 (9.3–16.1)	212 18.2 (16.0–20.4)	.016	204 17.8 (15.6–20.0)	8 42.1 (19.9–64.3)	.010

#### 3.2.2 Cause of death

The commonest causes of death in the surgical group were cardiovascular and KRT withdrawal, while gastrointestinal complications were the most common cause in the nonoperative group (Table S6).

#### 3.2.3 Nonfatal complications for all patients on KRT

Patients who had surgery during their presentation of acute cholecystitis experienced higher unadjusted rates of cardiac arrest (2.3 vs. 0.6%, *p* = .010), blood transfusions (17.5 vs. 9.1%, *p* = .001), dialysis access dysfunction (4.5 vs. 1.7%, *p* = .004), ICU admission (19.2 vs. 6.4%, *p* = .001), and longer hospital stays (9 vs. 5 days, *p* < .001, Table 2). There was no difference in the incidence of sepsis or pneumonia. Patients who underwent surgery had a lower 30‐day readmission rate, although this was not statistically significant (12.5 vs. 17.6%, *p* = .436, Table 2).

Using multivariable logistic regression, surgery was significantly associated with increased odds of the composite of cardiovascular complications (MI, CVA, or cardiac arrest: OR 2.08, 95% CI (1.13–3.81)), ICU admission (OR 3.51, 95% CI (2.41–5.10)), and blood transfusions (OR 2.29, 95% CI (1.60–3.27), Table 3).

**TABLE 3:** Unadjusted and adjusted odds ratios of postoperative outcomes in patients who underwent cholecystectomy.

	Overall		Chronic dialysis		Kidney transplant	
	Unadjusted	Adjusted	Unadjusted	Adjusted	Unadjusted	Adjusted
Myocardial infarction	1.18 (0.55–2.57)	1.28 (0.57–2.87)	1.14 (0.45–2.85)	1.32 (0.52–3.35)	1.37 (0.30–6.23)	1.39 (0.26–7.19)
Cardiac arrest	3.82 (1.37–10.60)	2.90 (0.95–8.90)	7.12 (2.07–24.52)	6.32 (1.75–22.74)	0.60 (0.62–5.85)	*
Sepsis	1.45 (0.94–2.23)	1.45 (0.91–2.30)	1.81 (1.14–2.88)	1.80 (1.11–2.92)	0.53 (0.15–1.92)	0.63 (0.12–3.19)
Pneumonia	1.21 (0.66–2.12)	1.10 (0.57–2.12)	1.02 (0.48–2.15)	1.08 (0.51–2.30)	1.86 (0.59–5.91)	1.33 (0.35–5.09)
Delirium	1.04 (0.44–2.47)	1.01 (0.41–1.09)	1.17 (0.43–3.20)*	1.24 (0.44–3.44)	0.72 (0.14–3.78)	0.81 (0.14–4.69)
Blood transfusion	2.11 (1.51–2.97)	2.29 (1.60–3.27)	2.65 (1.82–3.85)	2.69 (1.83–3.94)	1.14 (0.50–2.62)	1.12 (0.44–2.83)
Pulmonary oedema	1.00 (0.56–1.81)	1.08 (0.58–2.00)	1.38 (0.75–2.52)	1.31 (0.70–2.45)	–	–
Hyperkalemia	1.74 (1.02–2.94)	1.55 (0.89–2.73)	1.48 (0.81–2.73)	1.41 (0.75–2.63)	5.00 (1.26–19.70)	4.08 (0.91–18.34)
Dialysis access dysfunction	2.70 (1.38–5.28)	3.93 (1.92–8.02)	3.27 (1.64–6.54)	3.52 (1.74–7.12)	1.82 (0.11–29.40)	2.95 (0.14–66.73)
Intensive care unit admission	3.44 (2.42–4.90)	3.51 (2.41–5.10)	4.16 (2.79–6.19)	4.17 (2.76–6.28)	2.08 (0.95–4.62)	2.19 (0.89–5.40)
Readmission within 30 days	0.67 (0.24–1.85)	0.51 (0.15–1.76)	0.78 (0.50–1.22)	0.86 (0.55–1.36)	0.58 (0.15–2.16)	0.53 (0.24–1.15)
Any cardiac (myocardial infarction, stroke, and cardiac arrest)	1.96 (1.12–3.50)	2.08 (1.13–3.81)	2.27 (1.21–4.26)	2.42 (1.26–4.65)	1.46 (0.39–5.58)	0.96 (0.21–4.47)
Any infection (pneumonia, sepsis, surgical site infection)	1.51 (1.06–2.14)	1.49 (1.02–2.17)	1.82 (1.23–2.68)	1.80 (1.20–2.70)	0.96 (0.41–2.22)	0.83 (0.30–2.31)

^*^ Insufficient events

#### 3.2.4 Nonfatal outcomes by KRT modality

Patients on chronic dialysis undergoing surgery had higher odds of cardiac arrest (OR 6.32, 95% CI 1.75–22.74), blood transfusion (OR 2.69, 95% CI 1.83–3.94), access dysfunction (OR 3.52, 95% CI 1.74–7.12), and ICU admission (OR 4.17, 95% CI 2.76–6.28) compared to patients on chronic dialysis who had nonoperative management, whereas the odds of these events in kidney transplant recipients who underwent a cholecystectomy were indifferent compared to those who did not have surgery (Table 3).

#### 3.2.5 Readmissions within 12 months in the nonoperative group

There were 187 all‐cause readmissions among 119 patients (10.2%) with acute cholecystitis within 12 months. Sixty‐five patients (5.6%) who re‐presented to the emergency department with acute cholecystitis within 12 months of their initial episode underwent cholecystectomy during their subsequent visit, with a median time of 1.8 months [0.1–5.4] from the initial presentation.

Approximately, one in five patients (*n* = 238, 20.4%) in the nonsurgical group had a cholecystectomy as an elective surgery with a median time of 2.2 [1.0–4.3] months. Postoperative outcomes of patients having emergent cholecystectomy in a readmission and those having surgery performed electively are presented in Table S7.

#### 3.2.6 Twelve‐month survival

Cholecystectomy during the initial emergent presentation was associated with lower mortality at 12 months compared to those who did not undergo a cholecystectomy (12.7 vs. 18.2%, *p* = .016, Table 2). The higher survival associated with surgery remained in the multivariable cox‐proportional hazards model for patients having surgical intervention (HR 0.61, 95% CI 0.43–0.87) and in the Kaplan–Meier analysis (Figure 2b, log‐rank test: *p* = .010). When stratified by KRT modality, the association of better survival with surgery was also observed at 12 months in patients on chronic dialysis (HR 0.68, 95% CI 0.46–0.98), but not in kidney transplant recipients (HR 0.35, 95% CI 0.11–1.11) (Table S5).

## 4 DISCUSSION

The standard of treatment of acute cholecystitis is a cholecystectomy, yet this binational data linkage study spanning 15 years identified that most patients with kidney failure on KRT (87%) are treated via nonoperative means. This study showed that in patients on chronic KRT presenting with acute cholecystitis, both short‐term fatal and nonfatal outcomes were not significantly different between patients who undergo surgery and those that do not. However, the lack of definitive treatment (i.e., cholecystectomy) was associated with a higher 30‐day readmission rate and lower 12‐month survival.

Perioperative outcomes of patients who undergo cholecystectomy are poorly described,^9^, ^10^, ^11^ particularly in patients on KRT who are often multimorbid and have decreased physiological reserve, thereby heightening their risk of an adverse event following a general anesthetic.

This study showed that the 30‐day mortality rate was not significantly different between patients who underwent surgery and their nonsurgical counterparts (5.4% vs. 5.1%, *p* = .830). Not surprisingly, the postoperative mortality rate was higher than that reported in the general population (<2%).^12^, ^13^ It is conceivable that given the observational nature of the study, there is a selection bias for patients who undergo cholecystectomy. However, there were no significant differences in the demographics or prevalence of comorbidities between the groups, and in fact, the patients who underwent cholecystectomy had a longer KRT vintage (4.0 vs. 2.9 years, *p* < .001), which is associated with an increased risk of postoperative mortality.^2^ Patients who underwent cholecystectomy for acute cholecystitis also had a 32% lower risk of 12‐month mortality compared with patients who received nonoperative management. Moreover, the increased mortality observed in the nonsurgical group went beyond that expected to be associated with patients' preexisting disease. These findings were observed for patients on chronic dialysis, and a similar trend was observed for kidney transplant recipients. This in part may have been due to the reduced gallbladder‐related complications and readmissions following definitive surgery.^14^ A similar 12‐month survival benefit for older patients in the general population (>65 years) undergoing cholecystectomy for acute cholecystitis compared to those receiving nonoperative management has also been seen in large national observational studies in England and North America.^14^, ^15^

Patients in the operative group experienced a higher rate of cardiac arrest (2.3 vs. 0.6, *p* = .010), more frequent dialysis access dysfunction (4.5 vs. 1.7%, *p* = .004), longer hospital stay (9 vs. 5 days, *p* < .001), a twofold increase in blood transfusion requirement, and a threefold increased likelihood of ICU admission. The odds of these adverse events for patients on chronic dialysis remained similar in the secondary analysis excluding kidney transplant recipients. Patients on chronic dialysis have a number of risk factors predisposing them to increased morbidity including accelerated vascular calcification, mineral bone disease, anemia, increased oxidative stress, and impaired immunity.^16^, ^17^ Furthermore, the high rates of blood transfusions warrant mentioning as they have their own risks including transmission of blood‐borne pathogens, haemolytic reaction, volume overload, and immunosuppression.^18^

The absence of validated preoperative risk assessment tools makes it challenging to identify patients for whom the risk–benefit balance favors surgery.^19^ The Tokyo Guidelines 2018 (TG18) were developed to guide the clinical management of acute cholecystitis based on the severity of clinical presentation, along with other predictive factors like the Charlson Comorbidity Index (CCI) and the American Society of Anesthesiologists Physical Status Classification (ASA‐PS) score. TG18 recommends that severe cases (Grade III) be managed by expert surgeons with additional specialized training, increased use of intensive care services in the perioperative setting, and possibly transferring patients to specialized centers. However, the effectiveness of TG18 in patients on chronic dialysis and those with kidney transplants is not yet known. This limitation could not be assessed in the study due to the nature of the dataset, but it highlights an important avenue for future research, especially with further evidence building to suggest that centers with high operative volumes tend to have better outcomes following cholecystectomies, reinforcing the need for specialized care.^20^, ^21^

The higher rate of any cause 30‐day readmission (18.2 vs. 12.7, *p* = .436) was similar to that observed in patients aged greater than 65 years receiving initial nonoperative management who had a 30‐day readmission rate up to 21% compared to 2.4% in patients having a cholecystectomy.^15^, ^22^ It is plausible that, in the patients who did not undergo surgery for acute cholecystitis, the readmissions were related to morbidity from preexisting non‐biliary disease. Unlike other illnesses involving inflammation of abdominal viscera, such as diverticulitis, where some patients may never experience another episode, it is understandable that current treatment algorithms suggest initial medical management for diverticulitis unless complications develop.^23^ However, our findings suggest that 1 in 10 patients represented with acute cholecystitis within 12 months of their initial presentation. Interestingly, the postoperative outcomes of patients who underwent emergency cholecystectomy in a subsequent readmission were comparable to those of patients who underwent elective cholecystectomy after their initial presentation, suggesting that these patients may have benefited from surgery in their initial presentation itself, potentially avoiding unnecessary readmissions and excess healthcare costs.

This study has a number of strengths including its comprehensive population‐based design with complete inclusion of all eligible patients across Australia and New Zealand involving more than 11 million consecutive hospital admissions. Nonetheless, there are also important limitations which need to be acknowledged. Firstly, surgery may have been offered to healthier patients and therefore the risk estimates may have been an underestimation. Given the nature of the dataset, neither clinical nor biochemical parameters were available to determine the severity of the clinical presentation nor assess the patient's physiological status which may in turn have influenced the decision to operate and overall prognosis.^24^ The nature of the dataset also prevented effective propensity score matching and analysis. Secondly, the timing of surgery within the presentation (i.e., early vs. late) could not be determined, which is pertinent since early surgery (<24 h of presentation) has been associated with a lower prevalence of postoperative complications and shorter hospital stays.^25^ For instance, there may have been patients who were initially deemed for nonoperative management who later required it because they were clinically deteriorating, and therefore more unwell at the time of surgery thereby overinflating the short‐term postoperative risk estimates calculated. Thirdly, due to the dataset coding structure, the authors were unable to reliably ascertain if the surgeries were performed laparoscopically or if patients underwent open surgery; the former being the standard of care. Fourthly, surgery‐specific postoperative complications (e.g., bile duct injury) could not be ascertained. Additionally, advanced procedures, such as endoscopic transpapillary gallbladder drainage or endoscopic ultrasound‐guided gallbladder drainage, were not assigned codes in the ACHI version used to identify procedures, and therefore the proportion of patients estimated to have undergone biliary drainage was an underestimate. Finally, the consistency of administrative coding of clinical data between jurisdictions may have varied. However, all jurisdictions were required to adhere to the Australian Coding Standard, which enhanced the robustness and validity of the data sources.^4^, ^5^

## 5 CONCLUSION

Acute cholecystitis is associated with significant mortality and morbidity in patients on chronic KRT. Cholecystectomy in the initial presentation of acute cholecystitis was not associated with altered 30‐day mortality compared to nonoperative management, but was associated with increased morbidity. Furthermore, surgery was associated with better survival at 12 months. The certainty of evidence would be strengthened by the conduct of well‐designed pragmatic trials involving both clinical and patient‐reported outcomes. This will help better inform shared decision‐making by patients and clinicians regarding the optimal treatment strategy for patients on chronic KRT.

## FUNDING INFORMATION

This work was supported by the Royal Australasian College of Physicians [DP is the recipient of the Royal Australasian College of Physicians Jacquot Research Entry Scholarship 2019–2020], Metro South Health Research Support Scheme Small Project Grant 2018, and University of Queensland Infrastructure Grant 2017.

## CONFLICT OF INTEREST STATEMENT

DJ is a current recipient of an Australian National Health and Medical Research Council Leadership Investigator Grant. DJ has received consultancy fees, research grants, speaker's honoraria and travel sponsorships from Baxter Healthcare and Fresenius Medical Care, consultancy fees from Astra Zeneca, Bayer, and AWAK, speaker's honoraria from ONO and BI & Lilly, and travel sponsorships from Ono and Amgen. CH has received funding from Janssen and GlaxoSmithKline to her institution for trial steering committee roles and research grant support to her institution from Shire, Baxter, Fresenius, and Otsuka, and travel sponsorship from Otsuka. NB has received honoraria from Baxter, education and travel grants from Roche and Amgen, and Consultancy fees from Astra Zeneca and Vifor.

## Supporting information


Appendix S1.
