# Supplementary material for: Operative and nonoperative management of acute cholecystitis in patients on chronic kidney replacement therapy
Source: J Hepatobiliary Pancreat Sci. 2025 Mar 25;32(6):465–75. doi: 10.1002/jhbp.12133 (PMC12188168; doi:10.1002/jhbp.12133)
Supplement: Supplementary file 1 — Appendix S1. [file JHBP-32-465-s001.docx]

**Supplementary Table S1:** ICD-10AM codes for Acute cholecystitis

K8000 Calculus of gallbladder with acute cholecystitis without obstruction,

K8001 Calculus of gallbladder with acute cholecystitis with obstruction,

K8040 Calculus of bile duct with cholecystitis, unspecified, without obstruction,

K8041 Calculus of bile duct with cholecystitis, unspecified, with obstruction,

K8050 Calculus of bile duct without cholangitis or cholecystitis without obstruction,

K810 Acute cholecystitis,

K800 Calculus of gallbladder with acute cholecystitis

K8000 Calculus of gallbladder with acute cholecystitis, without mention of obstruction

K8001 Calculus of gallbladder with acute cholecystitis, with obstruction

K801 Calculus of gallbladder with other cholecystitis

K8010 Calculus of gallbladder with other cholecystitis, without mention of obstruction

K8011 Calculus of gallbladder with other cholecystitis, with obstruction

K8020 Calculus of gallbladder without cholecystitis, without mention of obstruction

K804 Calculus of bile duct with cholecystitis

K8040 Calculus of bile duct with cholecystitis, without mention of obstruction

K8041 Calculus of bile duct with cholecystitis, with obstruction

K8051 Calculus of bile duct without cholangitis or cholecystitis, with obstruction

K81 Cholecystitis

K810 Acute cholecystitis

K811 Chronic cholecystitis

K818 Other cholecystitis

K819 Cholecystitis, unspecified

K820 Obstruction of gallbladder

K822 Perforation of gallbladder

K829 Disease of gallbladder, unspecified

**Supplementary Table S2:** ACHI codes for cholecystectomy and cholecystostomy

3044300 Cholecystectomy,

3044500 Laparoscopic cholecystectomy,

3044800 Laparoscopic cholecystectomy with exploration of common bile duct via cystic duct,

3044900 Laparoscopic cholecystectomy with exploration of common bile duct via laparoscopic choledochotomy,

3045401 Cholecystectomy with choledochotomy,

3045500 Cholecystectomy with choledochotomy and biliary intestinal anastomosis

**Cholecystostomy**

3037505 Cholecystostomy

9209600: Irrigation of cholecystostomy or other biliary tube

**Supplementary Table S3:** ICD-10AM codes for comorbidities

**Diabetes Mellitus:**

E090 Impaired glucose regulation with peripheral angiopathy

E0901 Impaired glucose regulation with peripheral angiopathy without gangrene

E0902 Impaired glucose regulation with peripheral angiopathy with gangrene

E091 Impaired glucose regulation with features of insulin resistance

E092 Intermediate hyperglycaemia with kidney complication

E0921 Intermediate hyperglycaemia with incipient nephropathy

E0929 Intermediate hyperglycaemia with other specified kidney complication

E093 Intermediate hyperglycaemia with ophthalmic complication

E0931 Intermediate hyperglycaemia with background retinopathy

E0932 Intermediate hyperglycaemia with preproliferative retinopathy

E094 Intermediate hyperglycaemia with neurological complication

E0940 Intermediate hyperglycaemia with unspecified neuropathy

E0942 Intermediate hyperglycaemia with polyneuropathy

E095 Intermediate hyperglycaemia with peripheral angiopathy

E0951 Intermediate hyperglycaemia with peripheral angiopathy, without gangrene

E0952 Intermediate hyperglycaemia with peripheral angiopathy, with gangrene

E097 Intermediate hyperglycaemia with multiple complications

E0971 Intermediate hyperglycaemia with multiple microvascular complications

E0972 Intermediate hyperglycaemia with features of insulin resistance

E13 Other specified diabetes mellitus

E097 Intermediate hyperglycaemia with multiple complications

E098 Intermediate hyperglycaemia with unspecified complication

E099 Intermediate hyperglycaemia without complication

E10 Type 1 diabetes mellitus

E100 Type 1 diabetes mellitus with hyperosmolarity

E1000 Insulin-dependent diabetes mellitus with coma, not stated as uncontrolled

E1001 Type 1 diabetes mellitus with hyperosmolarity without nonketotic hyperglycaemic-hyperosmolar coma (NKHHC)

E1002 Type 1 diabetes mellitus with hyperosmolarity with coma

E101 Type 1 diabetes mellitus with acidosis

E1010 Insulin-dependent diabetes mellitus with ketoacidosis, not stated as uncontrolled

E1011 Type 1 diabetes mellitus with ketoacidosis, without coma

E1012 Type 1 diabetes mellitus with ketoacidosis, with coma

E1013 Type 1 diabetes mellitus with lactic acidosis, without coma

E1014 Type 1 diabetes mellitus with lactic acidosis, with coma

E1015 Type 1 diabetes mellitus with ketoacidosis, with lactic acidosis, without coma

E1016 Type 1 diabetes mellitus with ketoacidosis, with lactic acidosis, with coma

E102 Type 1 diabetes mellitus with kidney complication

E1020 Type 1 diabetes mellitus with renal complication, unspecified

E1021 Type 1 diabetes mellitus with incipient diabetic nephropathy

E1022 Type 1 diabetes mellitus with established diabetic nephropathy

E1023 Type 1 diabetes mellitus with advanced renal disease

E1029 Type 1 diabetes mellitus with other specified kidney complication

E103 Type 1 diabetes mellitus with ophthalmic complication

E1030 Type 1 diabetes mellitus with ophthalmic complication, unspecified

E1031 Type 1 diabetes mellitus with background retinopathy

E1032 Type 1 diabetes mellitus with preproliferative retinopathy

E1033 Type 1 diabetes mellitus with proliferative retinopathy

E1034 Type 1 diabetes mellitus with other retinopathy

E1035 Type 1 diabetes mellitus with advanced ophthalmic disease

E1036 Type 1 diabetes mellitus with diabetic cataract

E1039 Type 1 diabetes mellitus with other specified ophthalmic complication

E104 Type 1 diabetes mellitus with neurological complication

E1040 Type 1 diabetes mellitus with unspecified neuropathy

E1041 Type 1 diabetes mellitus with diabetic mononeuropathy

E1042 Type 1 diabetes mellitus with diabetic polyneuropathy

E1043 Type 1 diabetes mellitus with diabetic autonomic neuropathy

E1049 Type 1 diabetes mellitus with other specified neurological complication

E105 Type 1 diabetes mellitus with circulatory complication

E1050 Type 1 diabetes mellitus with circulatory complication, unspecified

E1051 Type 1 diabetes mellitus with peripheral angiopathy, without gangrene

E1052 Type 1 diabetes mellitus with peripheral angiopathy, with gangrene

E1053 Type 1 diabetes mellitus with diabetic cardiomyopathy

E1059 Type 1 diabetes mellitus with other specified circulatory complication

E106 Type 1 diabetes mellitus with other specified complication

E1060 Insulin-dependent diabetes mellitus with other specified complications, not stated as uncontrolled

E1061 Type 1 diabetes mellitus with specified diabetic musculoskeletal and connective tissue complication

E1062 Type 1 diabetes mellitus with specified skin and subcutaneous tissue complication

E1063 Type 1 diabetes mellitus with specified periodontal complication

E1064 Type 1 diabetes mellitus with hypoglycaemia

E1065 Type 1 diabetes mellitus with poor control

E1069 Type 1 diabetes mellitus with other specified complication

E107 Type 1 diabetes mellitus with multiple complications

E1070 Insulin-dependent diabetes mellitus with multiple complications, not stated as uncontrolled

E1071 Type 1 diabetes mellitus with multiple microvascular and other specified nonvascular complications

E1073 Type 1 diabetes mellitus with foot ulcer due to multiple causes

E108 Type 1 diabetes mellitus with unspecified complication

E1080 Insulin-dependent diabetes mellitus with unspecified complications, not stated as uncontrolled

E1081 Insulin-dependent diabetes mellitus with unspecified complications, stated as uncontrolled

E109 Type 1 diabetes mellitus without complication

E1090 Insulin-dependent diabetes mellitus without complications, not stated as uncontrolled

E1091 Insulin-dependent diabetes mellitus without complications, stated as uncontrolled

E11 Type 2 diabetes mellitus

E110 Type 2 diabetes mellitus with hyperosmolarity

E1100 Non-insulin-dependent diabetes mellitus with coma, not stated as uncontrolled

E1101 Type 2 diabetes mellitus with hyperosmolarity without nonketotic hyperglycaemic-hyperosmolar coma (NKHHC)

E1102 Type 2 diabetes mellitus with hyperosmolarity with coma

E111 Type 2 diabetes mellitus with acidosis

E1110 Non-insulin-dependent diabetes mellitus with ketoacidosis, not stated as uncontrolled

E1111 Type 2 diabetes mellitus with ketoacidosis, without coma

E1112 Type 2 diabetes mellitus with ketoacidosis, with coma

E1113 Type 2 diabetes mellitus with lactic acidosis, without coma

E1114 Type 2 diabetes mellitus with lactic acidosis, with coma

E1115 Type 2 diabetes mellitus with ketoacidosis, with lactic acidosis, without coma

E1116 Type 2 diabetes mellitus with ketoacidosis, with lactic acidosis, with coma

E112 Type 2 diabetes mellitus with kidney complication

E1120 Type 2 diabetes mellitus with renal complication, unspecified

E1121 Type 2 diabetes mellitus with incipient diabetic nephropathy

E1122 Type 2 diabetes mellitus with established diabetic nephropathy

E1123 Type 2 diabetes mellitus with advanced renal disease

E1129 Type 2 diabetes mellitus with other specified kidney complication

E113 Type 2 diabetes mellitus with ophthalmic complication

E1130 Type 2 diabetes mellitus with ophthalmic complication, unspecified

E1131 Type 2 diabetes mellitus with background retinopathy

E1132 Type 2 diabetes mellitus with preproliferative retinopathy

E1133 Type 2 diabetes mellitus with proliferative retinopathy

E1134 Type 2 diabetes mellitus with other retinopathy

E1135 Type 2 diabetes mellitus with advanced ophthalmic disease

E1136 Type 2 diabetes mellitus with diabetic cataract

E1139 Type 2 diabetes mellitus with other specified ophthalmic complication

E114 Type 2 diabetes mellitus with neurological complication

E1140 Type 2 diabetes mellitus with unspecified neuropathy

E1141 Type 2 diabetes mellitus with diabetic mononeuropathy

E1142 Type 2 diabetes mellitus with diabetic polyneuropathy

E1143 Type 2 diabetes mellitus with diabetic autonomic neuropathy

E1149 Type 2 diabetes mellitus with other specified neurological complication

E115 Type 2 diabetes mellitus with circulatory complication

E1151 Type 2 diabetes mellitus with peripheral angiopathy, without gangrene

E1152 Type 2 diabetes mellitus with peripheral angiopathy, with gangrene

E1153 Type 2 diabetes mellitus with diabetic cardiomyopathy

E1159 Type 2 diabetes mellitus with other specified circulatory complication

E116 Type 2 diabetes mellitus with other specified complication

E1160 Non-insulin-dependent diabetes mellitus with other specified complications, not stated as uncontrolled

E1161 Type 2 diabetes mellitus with specified diabetic musculoskeletal and connective tissue complication

E1162 Type 2 diabetes mellitus with specified skin and subcutaneous tissue complication

E1169 Type 2 diabetes mellitus with other specified complication

E117 Type 2 diabetes mellitus with multiple complications

E1170 Non-insulin-dependent diabetes mellitus with multiple complications, not stated as uncontrolled

E1171 Type 2 diabetes mellitus with multiple microvascular and other specified nonvascular complications

E1172 Type 2 diabetes mellitus with features of insulin resistance

E1173 Type 2 diabetes mellitus with foot ulcer due to multiple causes

E118 Type 2 diabetes mellitus with unspecified complication

E1180 Non-insulin-dependent diabetes mellitus with unspecified complications, not stated as uncontrolled

E1181 Non-insulin-dependent diabetes mellitus with unspecified complications, stated as uncontrolled

E119 Type 2 diabetes mellitus without complication

E1190 Non-insulin-dependent diabetes mellitus without complications, not stated as uncontrolled

E1191 Non-insulin-dependent diabetes mellitus without complications, stated as uncontrolled

E13 Other specified diabetes mellitus

E130 Other specified diabetes mellitus with hyperosmolarity

E1300 Other specified diabetes mellitus with coma, not stated as uncontrolled

E1301 Other specified diabetes mellitus with hyperosmolarity, without nonketotic hyperglycaemic-hyperosmolar coma (NKHHC)

E1302 Other specified diabetes mellitus with hyperosmolarity, with coma

E131 Other specified diabetes mellitus with acidosis

E1310 Other specified diabetes mellitus with ketoacidosis, not stated as uncontrolled

E1311 Other specified diabetes mellitus with ketoacidosis, without coma

E1312 Other specified diabetes mellitus with ketoacidosis, with coma

E1313 Other specified diabetes mellitus with lactic acidosis, without coma

E1314 Other specified diabetes mellitus with lactic acidosis, with coma

E1315 Other specified diabetes mellitus with ketoacidosis, with lactic acidosis, without coma

E1316 Other specified diabetes mellitus with ketoacidosis, with lactic acidosis, with coma

E132 Other specified diabetes mellitus with kidney complication

E1320 Other specified diabetes mellitus with renal complication, unspecified

E1321 Other specified diabetes mellitus with incipient diabetic nephropathy

E1322 Other specified diabetes mellitus with established diabetic nephropathy

E1323 Other specified diabetes mellitus with advanced renal disease

E1329 Other specified diabetes mellitus with other specified kidney complication

E133 Other specified diabetes mellitus with ophthalmic complication

E1330 Other specified diabetes mellitus with ophthalmic complication, unspecified

E1331 Other specified diabetes mellitus with background retinopathy

E1332 Other specified diabetes mellitus with preproliferative retinopathy

E1333 Other specified diabetes mellitus with proliferative retinopathy

E1334 Other specified diabetes mellitus with other retinopathy

E1335 Other specified diabetes mellitus with advanced ophthalmic disease

E1336 Other specified diabetes mellitus with diabetic cataract

E1339 Other specified diabetes mellitus with other specified ophthalmic complication

E134 Other specified diabetes mellitus with neurological complication

E1340 Other specified diabetes mellitus with unspecified neuropathy

E1341 Other specified diabetes mellitus with diabetic mononeuropathy

E1342 Other specified diabetes mellitus with diabetic polyneuropathy

E1343 Other specified diabetes mellitus with diabetic autonomic neuropathy

E1349 Other specified diabetes mellitus with other specified neurological complication

E135 Other specified diabetes mellitus with circulatory complication

E1350 Other specified diabetes mellitus with circulatory complication, unspecified

E1351 Other specified diabetes mellitus with peripheral angiopathy, without gangrene

E1352 Other specified diabetes mellitus with peripheral angiopathy, with gangrene

E1353 Other specified diabetes mellitus with diabetic cardiomyopathy

E1359 Other specified diabetes mellitus with other specified circulatory complication

E136 Other specified diabetes mellitus with other specified complication

E1360 Other specified diabetes mellitus with other specified complications, not stated as uncontrolled

E1361 Other specified diabetes mellitus with specified diabetic musculoskeletal and connective tissue complication

E1362 Other specified diabetes mellitus with specified skin and subcutaneous tissue complication

E1363 Other specified diabetes mellitus with specified periodontal complication

E1364 Other specified diabetes mellitus with hypoglycaemia

E1365 Other specified diabetes mellitus with poor control

E1369 Other specified diabetes mellitus with other specified complication

E137 Other specified diabetes mellitus with multiple complications

E1370 Other specified diabetes mellitus with multiple complications, not stated as uncontrolled

E1371 Other specified diabetes mellitus with multiple microvascular and other specified nonvascular complications

E1372 Other specified diabetes mellitus with features of insulin resistance

E1373 Other specified diabetes mellitus with foot ulcer due to multiple causes

E138 Other specified diabetes mellitus with unspecified complication

E1380 Other specified diabetes mellitus with unspecified complications, not stated as uncontrolled

E1381 Other specified diabetes mellitus with unspecified complications, stated as uncontrolled

E139 Other specified diabetes mellitus without complication

E1390 Other specified diabetes mellitus without complications, not stated as uncontrolled

E1391 Other specified diabetes mellitus without complications, stated as uncontrolled

E14 Unspecified diabetes mellitus

E140 Unspecified diabetes mellitus with hyperosmolarity

E1400 Unspecified diabetes mellitus with coma, not stated as uncontrolled

E1401 Unspecified diabetes mellitus with hyperosmolarity, without nonketotic hyperglycaemic-hyperosmolar coma (NKHHC)

E1402 Unspecified diabetes mellitus with hyperosmolarity, with coma

E141 Unspecified diabetes mellitus with acidosis

E1410 Unspecified diabetes mellitus with ketoacidosis, not stated as uncontrolled

E1411 Unspecified diabetes mellitus with ketoacidosis, without coma

E1412 Unspecified diabetes mellitus with ketoacidosis, with coma

E1413 Unspecified diabetes mellitus with lactic acidosis, without coma

E1414 Unspecified diabetes mellitus with lactic acidosis, with coma

E1415 Unspecified diabetes mellitus with ketoacidosis, with lactic acidosis, without coma

E1416 Unspecified diabetes mellitus with ketoacidosis, with lactic acidosis, with coma

E142 Unspecified diabetes mellitus with kidney complication

E1420 Unspecified diabetes mellitus with renal complication, unspecified

E1421 Unspecified diabetes mellitus with incipient diabetic nephropathy

E1422 Unspecified diabetes mellitus with established diabetic nephropathy

E1423 Unspecified diabetes mellitus with advanced renal disease

E1429 Unspecified diabetes mellitus with other specified kidney complication

E143 Unspecified diabetes mellitus with ophthalmic complication

E1430 Unspecified diabetes mellitus with ophthalmic complication, unspecified

E1431 Unspecified diabetes mellitus with background retinopathy

E1432 Unspecified diabetes mellitus with preproliferative retinopathy

E1433 Unspecified diabetes mellitus with proliferative retinopathy

E1434 Unspecified diabetes mellitus with other retinopathy

E1435 Unspecified diabetes mellitus with advanced ophthalmic disease

E1436 Unspecified diabetes mellitus with diabetic cataract

E1439 Unspecified diabetes mellitus with other specified ophthalmic complication

E144 Unspecified diabetes mellitus with neurological complication

E1440 Unspecified diabetes mellitus with unspecified neuropathy

E1441 Unspecified diabetes mellitus with diabetic mononeuropathy

E1442 Unspecified diabetes mellitus with diabetic polyneuropathy

E1443 Unspecified diabetes mellitus with diabetic autonomic neuropathy

E1449 Unspecified diabetes mellitus with other specified neurological complication

E145 Unspecified diabetes mellitus with circulatory complication

E1450 Unspecified diabetes mellitus with peripheral circulatory complications, not stated as uncontrolled

E1451 Unspecified diabetes mellitus with peripheral angiopathy, without gangrene

E1452 Unspecified diabetes mellitus with peripheral angiopathy, with gangrene

E1453 Unspecified diabetes mellitus with diabetic cardiomyopathy

E1459 Unspecified diabetes mellitus with other specified circulatory complication

E146 Unspecified diabetes mellitus with other specified complication

E1460 Unspecified diabetes mellitus with other specified complications, not stated as uncontrolled

E1461 Unspecified diabetes mellitus with specified diabetic musculoskeletal and connective tissue complication

E1462 Unspecified diabetes mellitus with specified skin and subcutaneous tissue complication

E1463 Unspecified diabetes mellitus with specified periodontal complication

E1464 Unspecified diabetes mellitus with hypoglycaemia

E1465 Unspecified diabetes mellitus with poor control

E1469 Unspecified diabetes mellitus with other specified complication

E147 Unspecified diabetes mellitus with multiple complications

E1470 Unspecified diabetes mellitus with multiple complications, not stated as uncontrolled

E1471 Unspecified diabetes mellitus with multiple microvascular and other specified nonvascular complications

E1471 Unspecified diabetes mellitus with multiple microvascular and other specified nonvascular complications

E1472 Unspecified diabetes mellitus with features of insulin resistance

E1473 Unspecified diabetes mellitus with foot ulcer due to multiple causes

E148 Unspecified diabetes mellitus with unspecified complication

E1480 Unspecified diabetes mellitus with unspecified complications, not states as uncontrolled

E1481 Unspecified diabetes mellitus with unspecified complications, stated as uncontrolled

E149 Unspecified diabetes mellitus without complication

E1490 Unspecified diabetes mellitus without complications, not stated as uncontrolled

E1491 Unspecified diabetes mellitus without complications, stated

**ISCHAEMIC HEART DISEASE:**

I210 Acute transmural myocardial infarction of anterior wall

I21 Acute myocardial infarction

I211 Acute transmural myocardial infarction of inferior wall

I212 Acute transmural myocardial infarction of other sites

I213 Acute transmural myocardial infarction of unspecified site

I214 Acute subendocardial myocardial infarction

I219 Acute myocardial infarction, unspecified

I22 Subsequent myocardial infarction

I220 Subsequent myocardial infarction of anterior wall

I221 Subsequent myocardial infarction of inferior wall

I228 Subsequent myocardial infarction of other sites

I229 Subsequent myocardial infarction of unspecified site

I23 Certain current complications following acute myocardial infarction

I230 Haemopericardium as current complication following acute myocardial infarction

I231 Atrial septal defect as current complication following acute myocardial infarction

I232 Ventricular septal defect as current complication following acute myocardial infarction

I233 Rupture of cardiac wall without haemopericardium as current complication following acute myocardial infarction

I234 Rupture of chordae tendineae as current complication following acute myocardial infarction

I235 Rupture of papillary muscle as current complication following acute myocardial infarction

I236 Thrombosis of atrium, auricular appendage, and ventricle as current complications following acute myocardial infarction

I238 Other current complications following acute myocardial infarction

I24 Other acute ischaemic heart diseases

I240 Coronary thrombosis not resulting in myocardial infarction

I241 Dressler's syndrome

I248 Other forms of acute ischaemic heart disease

I249 Acute ischaemic heart disease, unspecified

I200 Unstable angina

I201 Angina pectoris with documented spasm

I208 Other forms of angina pectoris

I209 Angina pectoris, unspecified

I250 Atherosclerotic cardiovascular disease, so described

I251 Atherosclerotic heart disease

I252 Old myocardial infarction

I253 Aneurysm of heart

I254 Coronary artery aneurysm and dissection

I255 Ischaemic cardiomyopathy

I256 Silent myocardial ischaemia

I258 Other forms of chronic ischaemic heart disease

I259 Chronic ischaemic heart disease, unspecified

I2511 Atherosclerotic heart disease, of native coronary artery

I2512 Atherosclerotic heart disease, of autologous bypass graft

I2513 Atherosclerotic heart disease, of nonautologous bypass graft

U821 Ischaemic heart disease

**CEREBROVASCULAR DISEASE**

I672 Cerebral atherosclerosis

I678 Other specified cerebrovascular diseases

I679 Cerebrovascular disease, unspecified

G459 Transient cerebral ischaemic attack, unspecified

G453Amaurosis fugax

I693 Sequelae of cerebral infarction

I610 Intracerebral haemorrhage in hemisphere, subcortical

I611 Intracerebral haemorrhage in hemisphere, cortical

I612 Intracerebral haemorrhage in hemisphere, unspecified

I613 Intracerebral haemorrhage in brain stem

I614 Intracerebral haemorrhage in cerebellum

I615 Intracerebral haemorrhage, intraventricular

I616 Intracerebral haemorrhage, multiple localised

I618 Other intracerebral haemorrhage

I619 Intracerebral haemorrhage, unspecified

I61 Intracerebral haemorrhage

I691 Sequelae of intracerebral haemorrhage

S0623 Multiple intracerebral and cerebellar haematomas

I630 Cerebral infarction due to thrombosis of precerebral arteries

I631 Cerebral infarction due to embolism of precerebral arteries

I632 Cerebral infarction due to unspecified occlusion or stenosis of precerebral arteries

I634 Cerebral infarction due to embolism of cerebral arteries

I635 Cerebral infarction due to unspecified occlusion or stenosis of cerebral arteries

I636 Cerebral infarction due to cerebral venous thrombosis, nonpyogenic

I638 Other cerebral infarction

I639 Cerebral infarction, unspecified

I63 Cerebral infarction

G463 Brain stem stroke syndrome (I60-I67+)

I64 Stroke, not specified as haemorrhage or infarction

I694 Sequelae of stroke, not specified as haemorrhage or infarction

I693 Sequelae of cerebral infarction

I679 Cerebrovascular disease, unspecified

**CHORNIC OBSTRCUTIVE AIRWAY DISEASE**

J440 Chronic obstructive pulmonary disease with acute lower respiratory infection

J441 Chronic obstructive pulmonary disease with acute exacerbation, unspecified

J448 Other specified chronic obstructive pulmonary disease

J449 Chronic obstructive pulmonary disease, unspecified

J43 Emphysema J431 Panlobular emphysema

J432 Centrilobular emphysema

J438 Other emphysema

J439 Emphysema, unspecified

J44 Other chronic obstructive pulmonary disease

U832 Chronic obstructive pulmonary disease

U831 Emphysema, without mention of chronic obstructive pulmonary disease

J410 Simple chronic bronchitis

J411 Mucopurulent chronic bronchitis

J47 Bronchiectasis

U834 Bronchiectasis, without mention of cystic fibrosis

J841 Other interstitial pulmonary diseases with fibrosis

J848 Other specified interstitial pulmonary diseases

J849 Interstitial pulmonary disease, unspecified

I270 Primary pulmonary hypertension

I272 Other secondary pulmonary hypertension

I278 Other specified pulmonary heart diseases

I28 Other diseases of pulmonary vessels

I288 Other specified diseases of pulmonary vessels

I289 Disease of pulmonary vessels, unspecified

G473 Sleep apnoea G4730 Sleep apnoea, unspecified

G4731 Central sleep apnoea syndrome

G4732 Obstructive sleep apnoea syndrome

G4733 Sleep hypoventilation syndrome

G4739 Other sleep apnoea

I270 Primary pulmonary hypertension

I272 Other secondary pulmonary hypertension

I278 Other specified pulmonary heart diseases

I28 Other diseases of pulmonary vessels

I288 Other specified diseases of pulmonary vessels

I289 Disease of pulmonary vessels, unspecified

J841 Other interstitial pulmonary diseases with fibrosis

J848 Other specified interstitial pulmonary diseases

J849 Interstitial pulmonary disease, unspecified

**PERIPHERAL VASCULAR DISEASE**

I700 Atherosclerosis of aorta

I701 Atherosclerosis of renal artery

I702 Atherosclerosis of arteries of extremities

I708 Atherosclerosis of other arteries

I709 Generalised and unspecified atherosclerosis

I738 Other specified peripheral vascular diseases

I739 Peripheral vascular disease, unspecified

I73 Other peripheral vascular diseases

I742 Embolism and thrombosis of arteries of upper extremities

I743 Embolism and thrombosis of arteries of lower extremities

I744 Embolism and thrombosis of arteries of extremities, unspecified

**HYPERTENSION**

I10 Essential (primary) hypertension

I11 Hypertensive heart disease

I110 Hypertensive heart disease with (congestive) heart failure

I119 Hypertensive heart disease without (congestive) heart failure

I12 Hypertensive kidney disease

I120 Hypertensive kidney disease with kidney failure

I129 Hypertensive kidney disease without kidney failure

I13 Hypertensive heart and kidney disease

I130 Hypertensive heart and kidney disease with (congestive) heart failure

I131 Hypertensive heart and kidney disease with kidney failure

I132 Hypertensive heart and kidney disease with both (congestive) heart failure and kidney failure

I139 Hypertensive heart and kidney disease, unspecified

I15 Secondary hypertension

I150 Renovascular hypertension

I151 Hypertension secondary to other kidney disorders

I152 Hypertension secondary to endocrine disorders

I158 Other secondary hypertension

I159 Secondary hypertension, unspecified

**HEART FAILURE/CARDIOMYOPATHY**

I500 Congestive heart failure

I501 Left ventricular failure

I509 Heart failure, unspecified

I130 Hypertensive heart and kidney disease with (congestive) heart failure

I132 Hypertensive heart and kidney disease with both (congestive) heart failure and kidney failure

I50 Heart failure

I110 Hypertensive heart disease with (congestive) heart failure

I119 Hypertensive heart disease without (congestive) heart failure

I420 Dilated cardiomyopathy

I421 Obstructive hypertrophic cardiomyopathy

I422 Other hypertrophic cardiomyopathy

I423 Endomyocardial (eosinophilic) disease

I424 Endocardial fibroelastosis

I425 Other restrictive cardiomyopathy

I426 Alcoholic cardiomyopathy

I427 Cardiomyopathy due to drugs and other external agents

I428 Other cardiomyopathies

I429 Cardiomyopathy, unspecified

I430 Cardiomyopathy in infectious and parasitic diseases classified elsewhere

I432 Cardiomyopathy in nutritional diseases

I438 Cardiomyopathy in other diseases classified elsewhere

**SLEEP APNOEA**

G473 Sleep apnoea

G4730 Sleep apnoea, unspecified

G4731 Central sleep apnoea syndrome

G4732 Obstructive sleep apnoea syndrome

G4733 Sleep hypoventilation syndrome

G4739 Other sleep apnoea

**SUPPLEMENTARY TABLE S4:** ICD-10AM codes for comorbidities

**MYOCARDIAL INFARCTION**

I210 Acute transmural myocardial infarction of anterior wall

I21 Acute myocardial infarction

I211 Acute transmural myocardial infarction of inferior wall

I212 Acute transmural myocardial infarction of other sites

I213 Acute transmural myocardial infarction of unspecified site

I214 Acute subendocardial myocardial infarction

I219 Acute myocardial infarction, unspecified

I22 Subsequent myocardial infarction

I220 Subsequent myocardial infarction of anterior wall

I221 Subsequent myocardial infarction of inferior wall

I228 Subsequent myocardial infarction of other sites

I229 Subsequent myocardial infarction of unspecified site

I23 Certain current complications following acute myocardial infarction

I230 Haemopericardium as current complication following acute myocardial infarction

I231 Atrial septal defect as current complication following acute myocardial infarction

I232 Ventricular septal defect as current complication following acute myocardial infarction

I233 Rupture of cardiac wall without haemopericardium as current complication following acute myocardial infarction

I234 Rupture of chordae tendineae as current complication following acute myocardial infarctionv

I235 Rupture of papillary muscle as current complication following acute myocardial infarction

I236 Thrombosis of atrium, auricular appendage, and ventricle as current complications following acute myocardial infarction

I238 Other current complications following acute myocardial infarction

I24 Other acute ischaemic heart diseases

I240 Coronary thrombosis not resulting in myocardial infarction

I241 Dressler's syndrome

I248 Other forms of acute ischaemic heart disease

I249 Acute ischaemic heart disease, unspecified

**CARDIAC ARREST**

I461 Sudden cardiac death, so described

I469 Cardiac arrest, unspecified

I460 Cardiac arrest with successful resuscitation

I46 Cardiac arrest

**DEEP VEIN THROMBOSIS**

I829 Embolism and thrombosis of unspecified vein

I828 Embolism and thrombosis of other specified veins

I801 Phlebitis and thrombophlebitis of femoral vein

I8020 Phlebitis and thrombophlebitis of other deep vessels of lower extremities

I8021 Phlebitis and thrombophlebitis of iliac vein

I8022 Phlebitis and thrombophlebitis of popliteal vein

I8023 Phlebitis and thrombophlebitis of tibial vein

I8042 Phlebitis and thrombophlebitis of deep vessels of upper extremities

**CEREBROVASCULAR ACCIDENT**

I630 Cerebral infarction due to thrombosis of precerebral arteries

I631 Cerebral infarction due to embolism of precerebral arteries

I632 Cerebral infarction due to unspecified occlusion or stenosis of precerebral arteries

I633 Cerebral infarction due to thrombosis of cerebral arteries

I634 Cerebral infarction due to embolism of cerebral arteries

I635 Cerebral infarction due to unspecified occlusion or stenosis of cerebral arteries

I636 Cerebral infarction due to cerebral venous thrombosis, nonpyogenic

I638 Other cerebral infarction

I639 Cerebral infarction, unspecified

I63 Cerebral infarction

I610 Intracerebral haemorrhage in hemisphere, subcortical

I611 Intracerebral haemorrhage in hemisphere, cortical

I612 Intracerebral haemorrhage in hemisphere, unspecified

I613 Intracerebral haemorrhage in brain stem

I614 Intracerebral haemorrhage in cerebellum

I615 Intracerebral haemorrhage, intraventricular

I616 Intracerebral haemorrhage, multiple localized

I618 Other intracerebral haemorrhage

I619 Intracerebral haemorrhage, unspecified

I620 Nontraumatic subdural haemorrhage

I621 Nontraumatic extradural haemorrhage

I629 Intracranial haemorrhage (nontraumatic), unspecified

I600 Subarachnoid haemorrhage from carotid siphon and bifurcation

I601 Subarachnoid haemorrhage from middle cerebral artery

I602 Subarachnoid haemorrhage from anterior communicating artery

I603 Subarachnoid haemorrhage from posterior communicating artery

I604 Subarachnoid haemorrhage from basilar artery

I605 Subarachnoid haemorrhage from vertebral artery

I606 Subarachnoid haemorrhage from other intracranial arteries

I607 Subarachnoid haemorrhage from intracranial artery, unspecified

I608 Other subarachnoid haemorrhage

I609 Subarachnoid haemorrhage, unspecified

G463 Brain stem stroke syndrome

I64 Stroke, not specified as haemorrhage or infarction

I694 Sequelae of stroke, not specified as haemorrhage or infarction

**PULMONARY EMBOLISM**

I269 Pulmonary embolism without mention of acute cor pulmonale

I26 Pulmonary embolism

I260 Pulmonary embolism with mention of acute cor pulmonale

**DEEP VEIN THROMBOSIS**

I829 Embolism and thrombosis of unspecified vein

I828 Embolism and thrombosis of other specified veins

I801 Phlebitis and thrombophlebitis of femoral vein

**PNEUMONIA**

J100 Influenza with pneumonia, other influenza virus identified

J110 Influenza with pneumonia, virus not identified

J120 Adenoviral pneumonia

J121 Respiratory syncytial virus pneumonia

J122 Parainfluenza virus pneumonia

J123 Human metapneumovirus pneumonia

J128 Other viral pneumonia

J129 Viral pneumonia, unspecifiedJ13 Pneumonia due to Streptococcus pneumoniae

J14 Pneumonia due to Haemophilus influenzae

J958 Other postprocedural respiratory disorders

J959 Postprocedural respiratory disorder, unspecified

J15 Bacterial pneumonia, not elsewhere classified

J150 Pneumonia due to Klebsiella pneumoniae

J151 Pneumonia due to Pseudomonas

J152 Pneumonia due to staphylococcus

J153 Pneumonia due to streptococcus, group B

J154 Pneumonia due to other streptococci

J155 Pneumonia due to Escherichia coli

J156 Pneumonia due to other (aerobic) Gram-negative bacteria

J157 Pneumonia due to Mycoplasma pneumoniae

J158 Other bacterial pneumonia

J159 Bacterial pneumonia, unspecified

J16 Pneumonia due to other infectious organisms, not elsewhere classified

J160 Chlamydial pneumonia

J168 Pneumonia due to other specified infectious organisms

J172 Pneumonia in mycoses

J958 Other postprocedural respiratory disorders

J168 Pneumonia due to other specified infectious organisms

J16 Pneumonia due to other infectious organisms, not elsewhere classified

J17 Pneumonia in diseases classified elsewhere

J170 Pneumonia in bacterial diseases classified elsewhere

J1171 Pneumonia in viral diseases classified elsewhere

J178 Pneumonia in other diseases classified elsewhere

J18 Pneumonia, organism unspecified

J180 Bronchopneumonia, unspecified

J181 Lobar pneumonia, unspecified

J188 Other pneumonia, organism unspecified

J189 Pneumonia, unspecified

J690 Pneumonitis due to food and vomit

J200 Acute bronchitis due to Mycoplasma pneumoniae

J851 Abscess of lung with pneumonia

J440 Chronic obstructive pulmonary disease with acute lower respiratory infection

J441 Chronic obstructive pulmonary disease with acute exacerbation, unspecified

**SUIRGICAL SITE INFECTION**

T814 Infection following a procedure, not elsewhere classified

T8141 Wound infection following a procedure

T8142 Sepsis following a procedure

T81 Complications of procedures, not elsewhere classified

T846 Infection and inflammatory reaction due to internal fixation device [any site]

T857 Infection and inflammatory reaction due to other internal prosthetic devices, implants and grafts

T8578 Infection and inflammatory reaction due to other internal prosthetic devices, implants and grafts

T818 Other complications of procedure, not elsewhere classified

T819 Unspecified complication of procedure

**SEPSIS**

A400 Sepsis due to streptococcus, group A

A401 Sepsis due to streptococcus, group B

A402 Sepsis due to streptococcus, group D

A403 Sepsis due to Streptococcus pneumoniae

A408 Other streptococcal sepsis

A409 Streptococcal sepsis, unspecified

A410 Sepsis due to Staphylococcus aureus

A412 Sepsis due to unspecified staphylococcus

A413 Sepsis due to Haemophilus influenzae

A414 Sepsis due to anaerobes

A4150 Sepsis due to unspecified Gram-negative organisms

A4151 Sepsis due to Escherichia coli [E. Coli]|

A4152 Sepsis due to Pseudomonas

A4158 Sepsis due to other Gram-negative organisms

A418 Other specified sepsis

A419 Sepsis, unspecified

A4900 Staphylococcal infection, unspecified site

A4901 Staphylococcus aureus infection, unspecified site

A491 Streptococcal infection, unspecified site

A499 Bacterial infection, unspecified

A40 Streptococcal sepsis

A411 Sepsis due to other specified staphylococcus

A415 Sepsis due to other and unspecified Gram-negative organisms

A41 Other sepsisA499 Bacterial infection, unspecified

A48 Other bacterial diseases, not elsewhere classified

A49 Bacterial infection of unspecified site

**MECHANICAL COPMPLICATION**

T812 Accidental puncture and laceration during a procedure, not elsewhere classified

T813 Disruption of operation wound, not elsewhere classified

T84 Vascular complications following a procedure, not elsewhere classified

T840 Mechanical complication of internal joint prosthesis

T841 Mechanical complication of internal fixation device of bones of limb

T842 Mechanical complication of internal fixation device of other bones

T844 Mechanical complication of other internal orthopaedic devices, implants and grafts

T843 Mechanical complication of other bone devices, implants and grafts

T848 Other complications of internal orthopaedic prosthetic devices, implants and grafts T849 Unspecified complication of internal orthopaedic prosthetic device, implant and graft

T853 Mechanical complication of other ocular prosthetic devices, implants and grafts

T854 Mechanical complication of breast prosthesis and implant

T855 Mechanical complication of gastrointestinal prosthetic devices, implants and grafts

T856 Mechanical complication of other specified internal prosthetic devices, implants and grafts

T858 Other complications of internal prosthetic devices, implants and grafts, not elsewhere classified

T8581 Other complications due to nervous system device, implant or graft

T8588 Other complications of internal prosthetic device, implant and graft, NEC

T8589

T873 Neuroma of amputation stump

T875 Necrosis of amputation stump

T876 Other and unspecified complications of amputation stump

T822 Mechanical complication of coronary artery bypass and valve grafts

T823 Mechanical complication of other vascular grafts

T828 Other specified complications of cardiac and vascular prosthetic devices, implants and grafts

T827 Infection and inflammatory reaction due to other cardiac and vascular devices, implants and grafts

T8282 Unspecified complication of cardiac and vascular prosthetic device, implant and graft

T829 Unspecified complication of cardiac and vascular prosthetic device, implant and graft

T825 Mechanical complication of other cardiac and vascular devices and implants

T8259

**DELIRIUM**

F050 Delirium not superimposed on dementia, so described

F051 Delirium superimposed on dementia

F058 Other delirium

F059 Delirium, unspecified

**PULMONDARY ODEMA**

J81 Pulmonary oedema

J951 Acute pulmonary insufficiency following thoracic surgery

J952 Acute pulmonary insufficiency following nonthoracic surgery

R601 Generalised oedema

R60 Oedema, not elsewhere classified

E877 Fluid overload

E87 Other disorders of fluid, electrolyte and acid-base balance

E878 Other disorders of electrolyte and fluid balance, not elsewhere classified

**HYPERKALEMIA**

E875 Hyperkalaemia

**DIALYSIS ACCESS INTERVENTION**

T8286 Aneurysm following insertion of cardiac and vascular prosthetic devices, implants and grafts

T8282 Embolism and thrombosis following insertion of cardiac and vascular prosthetic devices, implants and grafts

T8281 Haemorrhage and haematoma following insertion of cardiac and vascular prosthetic devices, implants and grafts

T8276 Infection and inflammatory reaction due to surgically created arteriovenous fistula and shunt

T8253 Mechanical complication of surgically created arteriovenous fistula and shunt

T8283 Pain following insertion of cardiac and vascular prosthetic devices, implants and grafts

T8284 Stenosis following insertion of cardiac and vascular prosthetic devices, implants and grafts

T8289 Other specified complications of cardiac and vascular prosthetic devices, implants and grafts (can include fibrosis)

3451500Thrombectomy of arteriovenous fistula (includes surgical declotting of AVF / transcatheter infusion of thrombolytic or other agent)

3451800 Repair of surgically created arteriovenous fistula (includes correction of stenosis / revision / superficialisation / patch graft)

3451801 Repair of prosthetic (graft) arteriovenous access device (includes correction of stenosis / revision / patch graft)

3530307 Open transluminal balloon angioplasty (includes correction / revision of arteriovenous fistula stenosis by open transluminal balloon angioplasty)

3530307 Percutaneous transluminal balloon angioplasty (includes correction / revision of arteriovenous fistula stenosis by percutaneous transluminal balloon angioplasty)

3412102 Interruption of feeding vessels of arteriovenous fistula of limb

34130000 Closure of surgically created arteriovenous fistula of limb

**Supplementary Table S5:** Multivariable Cox-proportional hazard model for 30-day mortality adjusted for age, gender, BMI, KRT vintage, modality, DM and IHD.

|  | **All (n=1520)** | | | **Chronic dialysis only (n=1202)** | | | **Kidney transplant only (n=318)** | | |
| --- | --- | --- | --- | --- | --- | --- | --- | --- | --- |
|  | **Hazard ratio** | **95%CI** | **P** | **Hazard ratio** | **95%CI** | **P** | **Hazard ratio** | **95%CI** | **P** |
| 30-day mortality | | | | | | | | | |
| **Cholecystectomy** | 1.33 | 0.77-2.30 | 0.313 | 1.40 | 0.78-2.54 | 0.261 | 1.15 | 0.25-5.35 | 0.860 |
| **Age** | 1.05 | 1.03-1.07 | <0.001 | 1.05 | 1.02-1.07 | <0.001 | 1.05 | 0.99-1.16 | 0.091 |
| **Male sex** | 0.69 | 0.42-1.13 | 0.143 | 0.66 | 0.40-1.12 | 0.124 | 0.69 | 0.14-3.39 | 0.653 |
| **BMI** | 0.97 | 0.93-1.11 | 0.224 | 0.97 | 0.93-1.01 | 0.162 | 0.89 | 0.74-1.08 | 0.243 |
| **KRT vintage** | 1.06 | 1.02-1.11 | 0.006 | 1.06 | 1.01-1.12 | 0.010 | 1.00 | 0.89-1.12 | 0.952 |
| **KRT modality (HD)** |  |  |  |  |  |  |  |  |  |
| **PD** | 1.90 | 1.06-3.39 | 0.030 | - | - | - | - | - | - |
| **HHD** | 1.15 | 0.34-3.88 | 0.827 | - | - | - | - | - | - |
| **Kidney transplant** | 0.55 | 0.22-1.34 | 0.190 | - | - | - | - | - | - |
| **DM** | 1.45 | 0.87-2.40 | 0.153 | 1.63 | 0.94-2.83 | 0.079 | 0.56 | 0.11-2.84 | 0.480 |
| **IHD** | 1.76 | 1.06-2.90 | 0.027 | 1.53 | 0.90-2.60 | 0.114 | 5.90 | 1.14-30.36 | 0.034 |
| 12-month mortality | | | | | | | | | |
| **Cholecystectomy** | 0.61 | 0.43-0.87 | 0.007 | 0.68 | 0.46-0.98 | 0.040 | 0.35 | 0.11-1.11 | 0.075 |
| **Age** | 1.02 | 1.01-1.04 | <0.001 | 1.02 | 1.00-1.03 | <0.001 | 1.09 | 1.04-1.13 | <0.001 |
| **Male sex** | 0.92 | 0.70-1.20 | 0.548 | 0.90 | 0.68-1.19 | 0.476 | 1.85 | 0.60-5.71 | 0.283 |
| **BMI** | 0.97 | 0.95-0.99 | 0.002 | 0.96 | 0.94-0.98 | <0.001 | 0.94 | 0.84-1.05 | 0.259 |
| **KRT vintage** | 1.04 | 1.01-1.06 | 0.004 | 1.03 | 1.01-1.06 | 0.014 | 0.98 | 0.92-1.06 | 0.728 |
| **KRT modality (HD)** |  |  |  |  |  |  |  |  |  |
| **PD** | 1.32 | 0.96-1.82 | 0.090 | - | - | - | - | - | - |
| **HHD** | 0.91 | 0.48-1.73 | 0.778 | - | - | - | - | - | - |
| **Kidney transplant** | 0.43 | 0.25-0.74 | 0.002 | - | - | - | - | - | - |
| **Subsequent cholecystectomy** | 0.16 | 0.08-0.34 | <0.001 | 0.14 | 0.06-0.33 | <0.001 | 0.17 | 0.04-0.78 | 0.023 |
| **DM** | 1.32 | 1.01-1.74 | 0.046 | 1.51 | 1.13-2.01 | 0.005 | 0.27 | 0.07-1.09 | 0.309 |
| **IHD** | 1.57 | 1.20-2.07 | 0.001 | 1.59 | 1.20-2.10 | 0.001 | 1.27 | 0.38-4.23 | 0.701 |

**Supplementary Table S6:** Cause of death (30-day mortality).

|  | **Surgery (n=19)** | **No surgery (n=59)** |
| --- | --- | --- |
| **Cardiovascular (%)** | 5 (26.3) | 16 (27.1) |
| **Infection (%)** | 3 (15.8) | 7 (11.9) |
| **Withdrawal (%)** | 5 (26.3) | 8 (13.6) |
| **Gastrointestinal (%)** | 4 (21.1) | 20 (33.9) |
| **Other (%)** | 2 (10.5) | 8 (13.6) |

**Supplementary Table S7**: Postoperative outcomes of patients who had an emergent cholecystectomy in a subsequent admission versus a subsequent elective cholecystectomy.

|  | **Operation in emergency readmission**  **N=65** | **Operation as elective**  **N=238** |
| --- | --- | --- |
| Death  N  % (95%CI) | 1 (1.5) | 3 (1.3) |
| Myocardial infarction | 1 (1.5) | 1 (0.4) |
| Stroke | 0 (0) | 0 (0) |
| Cardiac arrest | 0 (0) | 1 (0.4) |
| PE | 0 (0) | 0 (0) |
| DVT | 0 (0) | 1 (0.4) |
| Pneumonia | 2 (3.1) | 7 (2.9) |
| Surgical site infection | 2 (3.1) | 9 (3.8) |
| Sepsis | 2 (3.1) | 5 (2.1) |
| Delirium | 0 (0) | 3 (1.3) |
| Mechanical wound complication | 5 (7.7) | 11 (4.6) |
| Blood transfusion | 9 (13.8) | 19 (8.0) |
| Pulmonary oedema | 5 (7.7) | 3 (1.3) |
| Hyperkalaemia | 4 (6.2) | 4 (1.7) |
| Dialysis access dysfunction | 5 (7.7) | 7 (2.9) |
|  |  |  |
| Length of stay | 11 [6-19] | 3 [1-6] |
| ICU admission | 2 (3.1) | 20 (8.4) |
| Readmission within 30 days | 11 (16.9) | 5 (2.1) |

**Supplementary Figure S1:** Kaplan-Meier survival curve for 1-year survival following initial presentation with acute cholecystitis (Dialysis patients only).

Log rank test: 0.079

| N at risk | 0 | 0.2 | 0.4 | 0.6 | 0.8 | 1 |
| --- | --- | --- | --- | --- | --- | --- |
| No surgery | 960 | 883 | 843 | 817 | 791 | 767 |
| Surgery | 242 | 224 | 220 | 216 | 211 | 205 |

**Supplementary Figure S2:** Kaplan-Meier survival curve for 1-year survival following initial presentation with acute cholecystitis (Kidney transplant patients only).

Log rank test: 0.349

| N at risk | 0 | 0.2 | 0.4 | 0.6 | 0.8 | 1 |
| --- | --- | --- | --- | --- | --- | --- |
| No surgery | 205 | 193 | 189 | 187 | 186 | 186 |
| Surgery | 113 | 109 | 106 | 105 | 105 | 105 |
